# Angular Phenozaxine Ethers as Potent Multi-microbial Targets Inhibitors: Design, Synthesis, and *Molecular Docking* Studies

**DOI:** 10.3389/fchem.2017.00107

**Published:** 2017-11-28

**Authors:** Mercy A. Ezeokonkwo, Onyinyechi N. Ogbonna, Sunday N. Okafor, Evelyn U. Godwin-Nwakwasi, Fidelia N. Ibeanu, Uchechukwu C. Okoro

**Affiliations:** ^1^Department of Pure and Industrial Chemistry, University of Nigeria, Nsukka, Nigeria; ^2^Department of Chemistry, Evangel University, Akaeze, Nigeria; ^3^Department of Pharmaceutical and Medicinal Chemistry, University of Nigeria, Nsukka, Nigeria; ^4^Department of Chemistry, Gregory University, Uturu, Nigeria; ^5^School of General Studies, University of Nigeria, Nsukka, Nigeria

**Keywords:** diazabenzo[a]phenoxazin-5-one, benzo[a]phenoxazin-5-one, phenols, ether, molecular docking, multi-drug target

## Abstract

The reaction of diaza-5H-benzo[a]phenoxazin-5-one and 5H-benzo[a]phenoxazin-5-one with various phenols catalyzed by Pd/t-BuXPhos/K_3_PO_4_ system gave previously unknown ether derivatives (**7a–f** and **8a–f**) in good yields. UV-visible, FTIR, and ^1^H NMR data were used to confirm structures of the synthesized compounds. The parent compounds and the derivatives were screened *in-silico* for their drug-likeness and binding affinities to the microbial targets through molecular docking. Molinspiration software and AutoDock were used for the drug-likeness and docking studies, respectively. All the synthesized compounds showed strong drug-likeness. They also showed excellent binding affinities with glucosamine-6-phosphate synthase (2VF5), AmpC beta-lactamase (1KE4), and Lanosterol-14α-demethylase (3JUV), with compound 7e having the highest binding energies −9.5, −9.3, and −9.3 kcal/mol, respectively. These were found to be higher than the binding energies of the standard drugs. The binding energies of ciprofloxacin with 2VF5 and 1KE4 were −7.8 and −7.5 kcal/mol, respectively, while that of ketoconazole with 3JUV was −8.6 kcal/mol. The study showed that the synthesized compounds have multi-target inhibitory effects and can be very useful in multi-drug resistance cases. A 2D quantitative structural activity relationship (QSAR) model against target Glucosamine-6-phosphate synthase (2VF5) was developed using partial least squares regression (PLS) with good internal prediction (*R*^2^ = 0.7400) and external prediction (*R*^2^_ predicted = 0.5475) via Molecular Operating Environment (2014).

## Introduction

Angular phenoxazines are interesting class of heterocyclic compounds owing to their various ranges of applications as dyes (Mass et al., 2003) and drugs (Boothroyd and Clark, 1953).

**Scheme 1:**
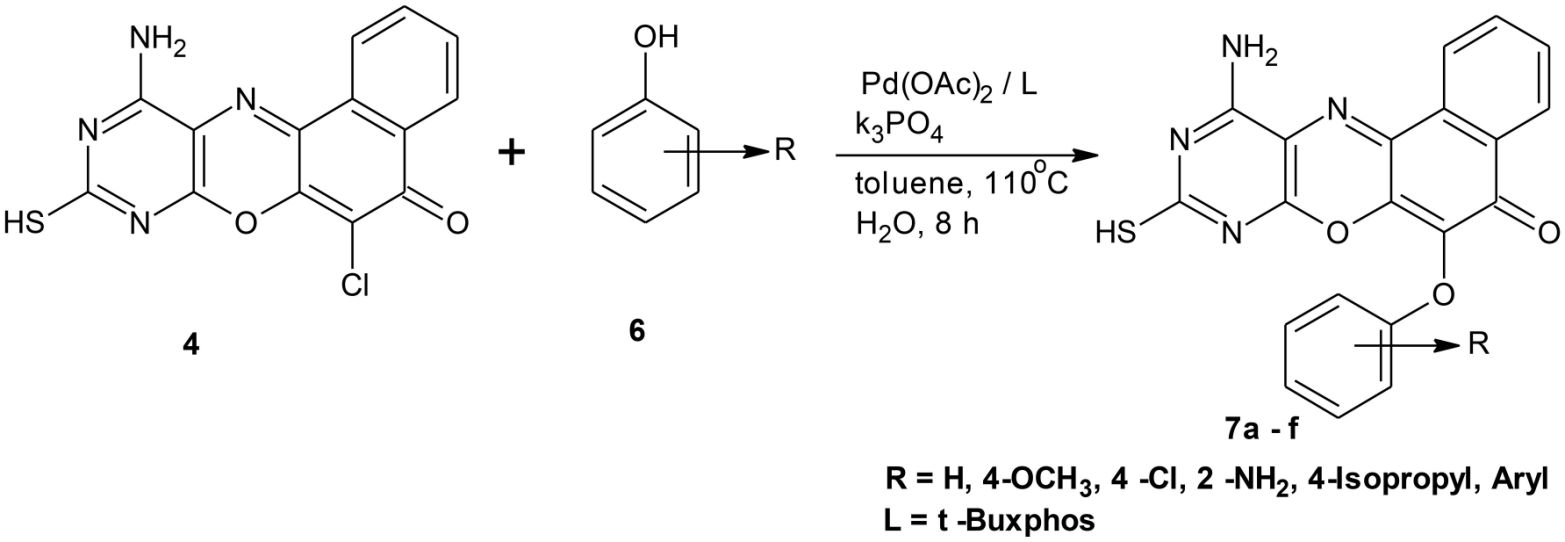
Synthesis of 11-amino-9-mercapto-6-chloro-8,10-diazabenzo[a]phenoxazin-5-one derivatives **7a–f**.

**Scheme 2:**
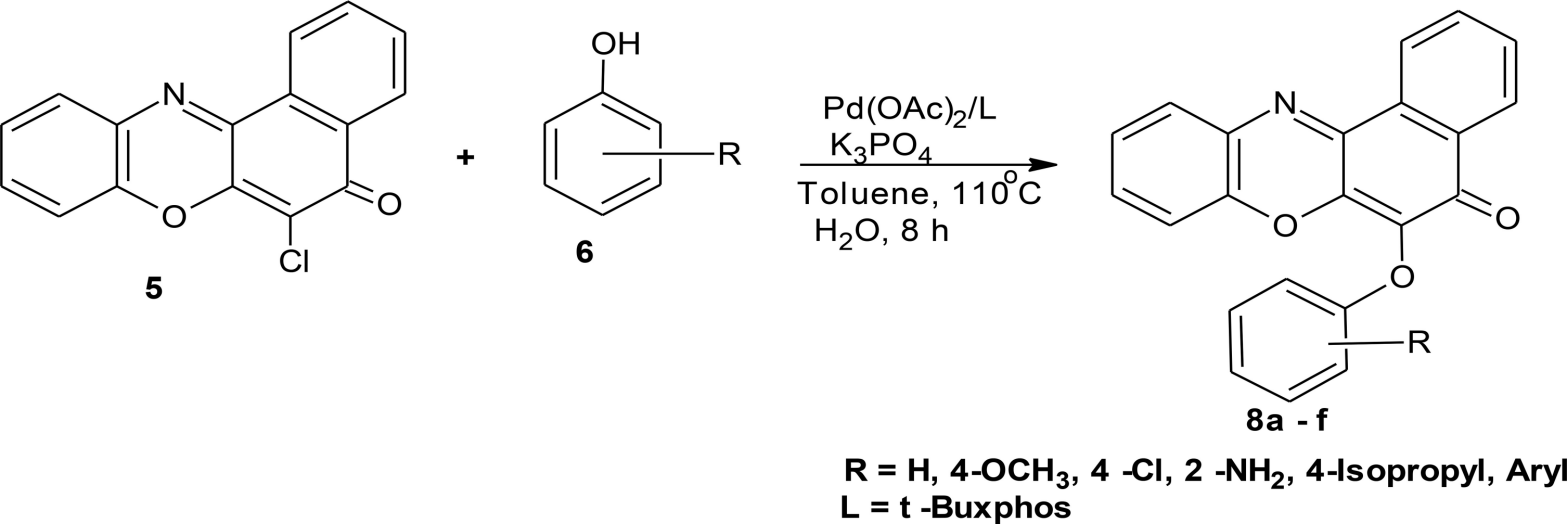
Synthesis of 6-chlorobenzo[a]phenoxazin-5-one derivatives **8a–f**.

Their utility in medicine includes as antitumour (Shimamoto et al., 2001), anticancer (Harton et al., 1993), antituberculosis (Boothroyd and Clark, 1953), and antibacterial (Chu Daniel, 1986) agents. The diaryl ether structural motifs are also very useful because of their wide applications in medicine and agriculture (Tomlin, 1994). They are basic structural units present in some bioactive compounds found in nature. For example, they are present in perrottetin (Bjon and Ulrich, 2004), riccardin B, and K-13 (Daniela et al., 2011). Mild aromatic C–O bond formation necessary in aryl ether synthesis is one of the difficult transformations in organic synthesis (Shelby et al., 2000). The conventional Ullmann coupling reaction used for diaryl ethers synthesis is limited because of the harsh reaction conditions, which are reflected in the high reaction temperature and the long reaction time (Ullmann, 1904). Palladium catalyzed coupling reaction has been used in recent times as a reliable tool for C–O bond formation (Hartwig, 2008; Martin and Buchwald, 2008), thereby providing a new and facile route to synthesis of diaryl ethers (Palucki et al., 1996, 1997; Attila et al., 1999; Torraca et al., 2001; Burgos et al., 2006). Attila and co-workers reported the successful coupling of a series of phenols with various aryl halides using a catalyst system consisting of palladium (II) acetate and di-t-BuXPhos to produce the corresponding diaryl ethers (Attila et al., 1999). Owing to the reported efficiency and importance of this reaction protocol coupled with its versatility and functional group tolerance, we have explored its application on complex heterocyclic substrates. The catalyst system was used in the synthesis of ether derivatives of diazaphenoxazine and related carbocyclic analog ring systems by reacting 11-amino-9-mercapto-6-chloro-8,10-diaza-5H-benzo[a]phenoxazin-5-one and 6-chloro-5H-benzo[a]phenoxazin-5-one with various phenol precursors (Schemes 1, 2). Although many works have been done in recent times in metal-catalyzed cross-coupling reactions involving phenols leading to ether formation, the utility of this protocol to complex heterocyclic compounds such as phenoxazines is not yet explored. Again, Okafor and coworkers (Okafor, 1986) reported the synthesis of 11-amino-9-mercapto-6-chloro-8,10-diazabenzo[a]phenoxazin-5-one and 6-chlorobenzo[a]phenoxazin-5-one but there has been relatively no report on the antimicrobial activity of these compounds and the synthesis of the ether derivatives. In view of these facts, we have investigated C-O cross-coupling reaction of diazabenzo[a]phenoxazin-5-one **4** and the carbocyclic analog **5** with varieties of phenols using palladium catalysis (Scheme 1, 2).

In addition, owing to rapid development of resistance by pathogenic organisms, and the need to synthesize and develop new and potent anti-microbial drugs, we carried out computational analysis on the synthesized compounds. Multidrug resistance (MDR) is said to occur when there is resistance or insensitivity of a microorganism to the administered antimicrobial agent, which was earlier sensitive to such agent (Singh, 2013; Popęda et al., 2014). WHO noted that these resistant microorganisms have the capacity to combat attack by antimicrobial drugs, thereby leading to therapeutic failure, which results in persistence and spreading of infections. Hiroshi (2009) has outlined many biochemical mechanisms of Multi-drug resistance. These include mutational alteration of the target protein, enzymatic inactivation of the drug, preventing drug access to targets, bypassing of the target, and acquisition of genes for less susceptible target proteins from other species. For example, aminoglycosides are inactivated by modifications that reduce the net positive charges on these polycationic antibiotics (Davies and Wright, 1997; Wright, 1999). The present work has been designed to look at three different targets and how the new synthesized compounds can interact with them to inhibit their normal biochemical processes. The targets are glucosamine-6-phosphate synthase, AmpC beta-lactamase, and Lanosterol-14a-demethylase (CYP51). The first two targets are derived from bacteria and the last from a fungus.

L-glutamine:D-fructose 6-phosphate amidotransferase or glucosamine-6-phosphate synthase (GlcN-6-P) (Duranda et al., 2008) with pdb code: 2VF5 is a very useful target in antimicrobial chemotherapy. 2VF5 catalyses the metabolism of hexosamine implicated in the biosynthesis of amino sugars (Zalkin, 1993; Massiere and Badet-Denisot, 1998; Zalkin and Smith, 1998). Its mechanism of action is to convert D-fructose-6-phosphate into d-glucosamine 6-phosphate (GlcN-6-P), which ultimately lead to the formation of uridine 5′-diphospho-N-acetyl-d-glucosamine (UDP-GlcNAc). UDP-GlcNAc is an important component of peptido glycan layer mostly found in the bacterial and fungal cell walls (Bearne and Blouin, 2000). Meanwhile, inactivation of GlcN-6-P synthase for a short period is very dangerous for fungal cells whereas a reduction in the amino sugar pool for such period does not constitute a treat to mammals. This is because mammalian cells have longer life span and the GlcN-6-P synthase has long half-lifetime. In addition, the mammalian gene encoding the enzyme GlcN-6-P synthase responds very fast (Milewski et al., 1986).

AmpC beta-lactamase (pdb code: 1KE4) is a group I, class C–lactamase enzyme. It is found in most Enterobacteriaceae, *Pseudomonas aeruginosa* and other non-fermenting gram-negative bacilli (Bush, 1995; Livermore, 1995). β-Lactamases hydrolyze and inactivate penicillins, cephalosporins antibiotics, and related molecules. This constitutes a major problem in development of drug resistance. β-lactamases, such as AmpC, are among the most problematic of these enzymes, and constitute serious public health concern. Therefore, there is an urgent need to find novel non-β-lactam-based inhibitors of these enzymes, which can stop the activities of the bacterial D,D-transpeptidases that are responsible for the last stage of peptidoglycan cross-linking.

Lanosterol-14a-demethylase (CYP51) (pdb code:3JUV) is an enzyme that plays a pivotal role in the biosynthesis of sterol in fungi (Vanden Bossche and Koymans, 1998). When this process is inhibited, it will result in the depletion of ergosterol and the accumulation of precursor 14a-methylated sterols. Georgopapadakou and Walsh (1996) and Lupetti et al. (2002) noted that this development will disrupt the structure of the plasma membrane and make it more vulnerable to further damage.

## Materials and methods

### Experimental

#### Reagents and apparatus

The phenoxazine ether derivatives were prepared based on the adoption of method by Attila et al. (1999). The reagents used in this work were of analytical grades, and were bought from Sigma Aldrich chemical company, Germany and Fluka Chemical Company. They were used as supplied with no additional purification. Fisher-Johns melting points apparatus was used for the determination of the melting points of the synthesized compounds, and the figures were uncorrected. UV/visible, IR, and ^1^H NMR spectroscopy were used to characterize the compounds. UV-visible spectra were determined in water on a JENWAY 6645 UV/VIS spectrophotometer. The absorption maxima are reported in nanometers, and the log ε-values are indicated in the parenthesis. Infrared spectra in wave numbers (cm^−1^) were determined on an alpha Bruker spectrophotometer. Nuclear magnetic resonance (^1^H NMR) spectra were determined using a Jeol 7000MH spectrometer at University of New Castle, London, United Kingdom. Chemical shifts were recorded on the delta (δ) scale. Interpretation of spectral data was done with reference to literature (Mohan, 2000).

### Synthesis

#### General procedures for Pd-catalyzed coupling of angular diaza-5H-benzo[a]phenoxazine and related carbocyclic analog with phenols

An oven dried three-necked flask was cooled to room temperature under a nitrogen flow. Into the flask was introduced palladium acetate (4.5 mg, 0.02 mmol, 2.0 mol%), t-BuXphos (0.03 mmol, 3.0 mol%), potassium phosphate (424.0 mg, 2.00 mmol), phenol (1.20 mmol), and phenoxazine compound (1.00 mmol). The flask was covered and made inert with nitrogen. Through the septum, toluene (3 ml) was introduced, and the flask made air-tight using a teflon screw cap. The reaction mixture was heated to 100–110°C and allowed to stir for 5–10 h while being monitored with thin layer chromatography. At the end of the reaction period, the mixture was cooled to room temperature and ethyl acetate (40 ml) added to it. The crude product was obtained through filtration, and subsequently recrystallized from a mixture of ethanol and water (2:1) to afford the pure compounds (**7a–f** and **8a–f**), respectively.

#### 11-amino-9-mercapto-6-(4-methoxyphenoxy)-8,10-diaza-5H-benzo[a]phenoxazin-5-one 7a

The general procedure was used to convert 11-amino-9-mercapto-8,10-diaza-5H-benzo[a]phenoxazin-5-one and methoxyphenol to the title compound **7a** in 8 h. Compound **7a** was gotten as dark brown solid after recrystallization from ethanol-water mixture (2:1). Yield 96%. mp >360°C. UV (H_2_O): λ_max_ (log ε_max_) 224 (3.7751), 390 (3.1940) nm. IR (neat, cm^−1^): 3203 (N–H, intramolecular H-bond with C = O), 1636 (C = O), 1581, 1501 (C = N, C = C), 1324 (C–N), 1267 (Asymmetric C–O–C stretch), 1059 (symmetric C–O–C stretch), 616 (C–S). ^1^H NMR (DMSO-d_6_, ppm): δ 3.65 (s, 1H, SH), 3.66 (s, 3H, OCH_3_), 5.47 (s, 2H, NH_2_), 6.67–6.73 (d, 2H, C-2, and C-6 of phenoxy), 7.52–7.77 (d, 2H, C-3, and C-5 of Phenoxy), 7.84–8.98 (s, 4H, Ar-H).

#### 11-amino-9-mercapto-6-(phenoxy)-8,10-diaza-5H-benzo[a]phenoxazin-5-one 7b

General procedure was used to convert 11-amino-9-mercapto-8,10-diaza-5H-benzo[a]phenoxazin-5-one and phenol in 10 h to 740 mg (96%) of the title compound **7b**. The compound was gotten as a dark brown solid after recrystallization from ethanol-water mixture (2:1). mp >360°C. UV (H_2_O): λ_max_ (log ε_max_) 251 (3.1638), 481 (2.4082) nm. IR (neat, cm^−1^): 3183 (N–H, intramolecular H-bond with C = O), 1632 (C = O), 1581, 1513 (C = N, C = C), 1378 (C–N), 1264, 1060 (C–O–C stretch), 616 (C–S). ^1^HNMR (DMSO-d_6_, ppm): δ 3.42 (s, 1H, SH), 5.48 (s, 2H, NH_2_), 6.75–6.79 (d, 1H, C2, and C6 of phenoxy), 7.16–7.17 (t, 3H, C3, C4, and C5 of phenoxyl), 7.77–8.52 (s, 4H, Ar-H).

#### 11-amino-9-mercaptho-6-(4-chlorophenoxy)-8,10-diaza-5H-benzo[a]phenoxazin-5-one 7c

General procedure was used to convert 11-amino-9-mercapto-8,10-diaza-5H-benzo[a]phenoxazin-5-one and 4-chlorophenol in 8 h to 630 mg (75%) of the title compound **7c**. The compound was a dark brown solid after recrystallization from ethanol-water mixture (2:1). mp > 360°C. UV (H_2_O): λ_max_ 258 (2.5340), 383 (2.1987), 484 (1.8513) nm. IR (neat, cm^−1^): 3190 (N–H, intramolecular H-bond with C = O), 1635 (C = O), 1580, 1501 (C = N, C = C), 1266 (C–N), 1222, 1060 (C–O–C), 780 (C–Cl), 616 (C–S). ^1^HNMR(δ): 3.65 (s, 1H, SH), 5.47 (s, 2H, NH_2_), 6.77–6.82 (d, 1H, C2, and C6 of phenoxy), 7.16–7.20 (d, 2H, C3, and C5 of phenoxy), 7.67–8.56 (s, 4H, Ar–H).

#### 11-amino-9-mercapto-6-(2-aminophenoxy)-8,10-diaza-5H-benzo[a]phenoxazin-5-one 7d

General procedure was used to convert 11-amino-9-mercapto-6-chloro-8,10-diaza-5H-benzo[a]phenoxazin-5-one and 2-aminophenol in 10 h to 736 mg (91%) of the title compound **7d**. The compound was obtained as a dark brown solid after recrystallization from ethanol-water mixture (2:1). Mp > 360°C. UV (H_2_O): λ_max_ 388 (2.8007) nm. IR (neat, cm^−1^): 3205 (N–H, intramolecular H-bond with C = O), 1631 (C = O), 1582, 1503 (C = N, C = C), 1306 (C–N), 1210, 1059 (C–O–C), 616 (C-S). ^1^HNMR (δ): 3.65 (s, 1H, SH), 5.46, 5.99 (s, 2H, 2NH_2_), 6.38 (d, 1H, C3 of phenoxy), 6.84–7.06 (t, 2H, C5, and C6 of phenoxy), 7.15–7.18 (t. 1H, C4 of phenoxy), 7.25–8.96 (s, 4H. Ar–H).

#### 11-amino-9-mercapto-6-(4-isopropylphenoxy)-8,10-diaza-5H-benzo[a]phenoxazin-5-one 7e

General procedure was used to convert 11-amino-9-mercapto-6-chloro-8,10-diaza-5H-benzo[a]phenoxazin-5-one and 4-isopropylpehnol in 7 h to 826 mg (96%) of the title compound **7e**. The compound was a dark brown solid after recrystallization from ethanol-water mixture (2:1). mp > 360°C. UV (H_2_O): λ_max_ 263 (3.1541), 379 (2.7067), 490 (2.3284) nm. IR (neat, cm^−1^): 3175 (N–H, intramolecular H-bond with C = O), 1635 (C–O), 1564, 1513 (C = N, C = C), 1337 (C–N), 1230, 1058 (C–O–C), 616 (C–S). ^1^HNMR (δ): 1.62, 1.63 (d, 6H. 2CH_3_), 3.28 (s, 1H, SH), 3.29 (m, 1H, methine CH), 5.54 (s, 1H, NH_2_) 6.67–6.71 (d, 1H, C2, and C6 of phenoxy), 7.52–7.66 (d, 2H, C3, and C5 of phenoxy), 7.77–8.38 (s, 4H. Ar–H).

#### 11-amino-9-mercapto-6-(naphthoxy)-8,10-diaza-5H-benzo[a]phenoxazin-5-one 7f

General procedure was used to convert 11-amino-9-mercapto-6-chloro-8,10-diaza-5H-benzo[a]phenoxazin-5-one and 1-naphthol in 10 h to 836 mg (99%) of the title compound **7f**. The compound was a dark brown solid after recrystallization from ethanol–water mixture (2:1). mp >360°C. UV (H_2_O): λ_max_ 217 (3.8476), 385 (3.1113) nm. IR (neat, cm^−1^): 3190 (N–H, intramolecular H-bond with C = O), 1640 (C = O), 1581, 1513 (C = N, C = C), 1327 (C–N), 1226, 1060 (C–O–C), 617 (C–S). ^1^HNMR (δ): 2.53 (s, 1H, SH), 5.47 (s, 1H, NH_2_), 6.67 (d, 1H, C_2_-H of naphthyl), 6.92–7.38 (m, 4H, C_3_-, C_4_-, C_6_-, and C_7_-H of naphthyl), 7.65–7.86 (m, 5H, C_5_-H of naphthoxy, and Ar–H), 8.47 (s, 1H, C8-H of naphthyl).

### 6-(4-methoxy phenoxy)-5H-benzo[a]phenoxazin-5-one 8a

General procedure was used to convert 6-chloro-5H-benzo[a]phenoxazin-5-one and methoxyphenol in 8 h to 710 mg (96%) of the title compound **8a**. The compound was a black solid after recrystallization from ethanol-water mixture (2:1). mp <360°C. UV (H_2_O): λ_max_ 269 (1.8261), 389(2.5977), 456 (2.5416) nm. IR (neat, cm^−1^): 3163 (intramolecular H-bond with C = O), 1626 (C = O), 1582 (C = N, C = C), 1208, 1060 (C–O–C). ^1^H NMR (δ): 3.37 (s, 3H, OCH_3_), 6.73–6.95 (m, 4H, phenoxy), 6.99–7.29 (m, 4H. Ar–H), 7.41–8.85 (s, 4H, Ar–H).

### 6-phenoxy-5H-benzo[a]phenoxazin-5-one 8b

General procedure was used to convert 6-chloro-5H-benzo[a]phenoxazin-5-one and phenol in 10 h to 750 mg (94%) of the title compound **8b**. The compound was a black solid after recrystallization from ethanol-water mixture (2:1). mp >360°C. UV (H_2_O): λ_max_ 270 (2.7050), 390 (2.6160), 490 (2.3979). IR (neat, cm^−1^): 3181(intramolecular H-bond with C = O), 1631 (C = O), 1563 (C = C, C = N), 1265, 1058 (C–O–C). ^1^H NMR (δ): 6.53–6.56 (d, 1H, C2, C4, and C6 of phenoxy), 6.88–7.03 (t, 2H, C3, and C5 of phenoxy) 7.17–7.53 (m, 4H, Ar–H), 7.67–8.53 (s, 4H, Ar–H).

### 6-(4-chlorophenoxy)-5H-benzo[a]phenoxazin-5-one 8c

General procedure was used to convert 5-chloro-5H-benzo[a]phenoxazin-5-one and 4-chlorophenol in 8 h to 770 mg (46%) of the title compound **8c**. The compound was obtained as a black solid after recrystallization from ethanol-water mixture (2:1). mp. >360°C. UV (H_2_O): λ_max_ 265 (2.7753), 387 (2.6284) nm. IR (neat, cm^−1^), 3172 (intramolecular H-bond with C = O), 1631 (C = O), 1563 (C = C, (C = N), 1263, 1059 (C–O–C). ^1^H NMR (δ): 6.78–6.79 (d, 2H, C2, and C6 of phenoxy), 7.15–7.17 (t, 2H, C3, and C5 of phenoxy), 7.25–7.50 (m, 4H, Ar–H), 7.87–8.68 (1, 4H, Ar–H).

### (2-aminophenoxy)-5H-benzo[a]phenoxazin-5-one 8d

General procedure was used to convert 6-chloro-5H-benzo[a]phenoxazin-5-one and 2-aminophenol in 10 h to 682 mg (94%) of the title compound **8d**. The compound was a black solid after recrystallization from ethanol-water mixture (2:1). mp > 360°C. UV (H_2_O): λ_max_ 234 (2.4216), 384 (2.1644), 493 (1.9684). IR (neat, cm^−1^): 3197 (N–H, intramolecular H-bond with C = O), 1632 (C = O), 1561(C = N), 1459 (C = C), 1323 (C–N), 1269, 1065 (C–O–C). ^1^H**NMR** (δ): 6.37 (s, 2H, NH_2_), 6.38 (d, 1H, C_3_-H of phenoxy), 6.77–6.78 (d, 1H,C_5_-H of phenoxy), 7.17–7.18 (t, 1H, C_6_-H of penoxy), 7.24–7.26 (t, 1H, C_4_-H of phenoxy), 7.39 (d, 1H, Ar–H), 7.40–7.41 (t, 1H, Ar–H), 7.45–7.51(m, 2H, Ar–H), 7.61–8.25 (m, 4H, Ar–H).

### 6-(4-isopropylphenoxy)-5H-benzo[a]phenoxazin-5-one 8e

General procedure was used to convert 6-chloro-5H-benzo[a]phenoxazin-5-one and 4-isopropylphenol in 7 h to 765 mg (98%) of the title compound **8e**. The compound was a black solid after recrystallization from ethanol-water mixture (2:1). mp >360°C. UV (H_2_O): λ_max_ 260 (3.2448), 384 (2.9294), 485(2.8859) nm. IR (neat, cm^−1^): 3194 (intramolecular H-bond with C = O), 1641 (C = O), 1563(C = N, C = C), 1266, 1059 (C–O–C). ^1^H NMR (δ): 1.61 (d, 6H, 2CH_3_), 2.86 (m, 1H, methane–H), 6.66–6.68 (d, 2H, C_2_, and C_6_ of phenoxy), 6.99–7.00 (m, 2H, C_3_, and C_5_ of phenoxy), 7.17–7.24 (m, 4H, Ar-H), and 0.55–7.91 (m, 4H, Ar–H).

### 6-(1-naphthoxy)-5h-benzo[a]phenoxazin-5-one 8f

General procedure was used to convert 6-chloro-5H-benzo[a]phenoxazin-5-one and 1-naphthol in 10 h to 520 mg (68%) of the title compound **8f**. The compound was a black solid after recrystallization from ethanol-water mixture (2:1). mp > 360°C. UV (H_2_O): λ_max_ 1263 (3.0078), 385 (2.4314) nm. IR (neat, cm^−1^): 3171 (intramolecular H-bond with C = O) 1626 (C = O), 1585 (C = C, C = N), 1234, 1059 (C–O–C). ^1^H NMR (δ): 6.37 (d, 1H, C_2_-H of naphthoxy), 6.62–6.87 (m, 4H, C_3_-, C_4_-, C_6_-, and C_7_-H of naphthoxy), 7.18–7.54 (m, 4H, Ar–H), 7.67–8.7 (m, 5H, Ar–H, andC_5_ of naphthoxy).

### *In-silico* physicochemical evaluation for drug-likeness

The physicochemical properties, including molecular weight, Log P, hydrogen bond donor (HBD), hydrogen bond acceptor, total polar surface, number of rotatable hydrogen, and volume, of the synthesized compounds were studied using Molinspiration Chemoinformatics softwares. The drug-likeness was evaluated using Lipinski's rule of five.

### Molecular docking studies

To have a better understanding on the binding modes of the synthesized derivatives (**7a–f and 8a–f**); the docking studies were performed. The 3D structures of the three bacteria target proteins were obtained from the Protein Data Bank. They were Glucosamine-6-phosphate synthase (PDB code: **2VF5)** at resolution of 2.9 Å (Mouilleron et al., 2008); AmpC beta-lactamase (PDB code: **1KE4**) at resolution of 1.72 Å (Powers and Shoichet, 2002), and Lanosterol 14-alpha-demethylase (PDB code: **3JUV)** at resolution of 3.12 Å (Strushkevich et al., 2013). We used Discovery Studio Visualizer 4.1 in the preparations of the proteins: the needed chains were selected and multiple ligands and non-protein parts were deleted. The 2D structures of the ligands were drawn using ACD/ChemSketch 2015 version and the 3D structures generated by the same software (ACD/ChemSketch, 2015). OpenBabel GUI version 2.3.2 (O'Boyle et al., 2011) and AutoDock were used to convert the pdb file format to pdbqt. Molecular docking process was done using AutoDock Tools 1.5.6 and AutoDock Vina version 1.1.2 (downloaded from http://autodock.scripps.edu). The docking results were analyzed and visualized with Pymol version 1.7.4.4.

### QSAR studies

#### Dataset preparation

A total of 100 known 2VF5 inhibitors were collected from various published literatures based on their structural diversity and activity coverage. The activity data for all compounds were taken from different scientific groups in terms of inhibitory activity (MIC μg/ml) (Jasmine et al., 2012; Shruthi et al., 2012; Jayanna et al., 2013; Hareesh et al., 2015; Preeti et al., 2015; Talavara et al., 2016). Fifty compounds out of hundred, were selected as a training set based following criteria that will produce a good quantitative structural activity relationship (QSAR) model. The biological activity for 2VF5 inhibitors ranged between 3.13 and 125 μg/ml.

#### Structure optimization

All the chemical structures were drawn using ChemSketch software. MOE software (MOE, 2014) was used for optimization and to check for any distortion in the bond angles and bond lengths. Energy minimization of all chemical structures was performed using molecular mechanism force field (MMFF94x) found in MOE. This software was also used for QSAR analysis.

#### Selection of relevant descriptors

A total of nine descriptors (as shown in Table 1) were chosen using the QuaSAR_Contingency Analysis in MOE so that we can get more robust and informative descriptors, and also to avoid the phenomenon of overfitting.

**Table 1:** QSAR descriptors.

**S/N**	**Descriptor**	**Description**
1	Weight	Molecular weight (including implicit hydrogens) in atomic mass units
2	logP(o/w)	Log of the octanol/water partition coefficient (including implicit hydrogens)
3	TPSA	Polar surface area (Å^2^)
4	b_rotN	Number of rotatable bonds
5	b_ar	Number of aromatic bond
6	Rings	The number of rings
7	a_don	Number of hydrogen bond donor atoms
8	a_acc	Number of hydrogen bond acceptor atoms
9	AM1_dipole	The dipole moment

## Results and discussion

### Chemistry

**Scheme 3:**
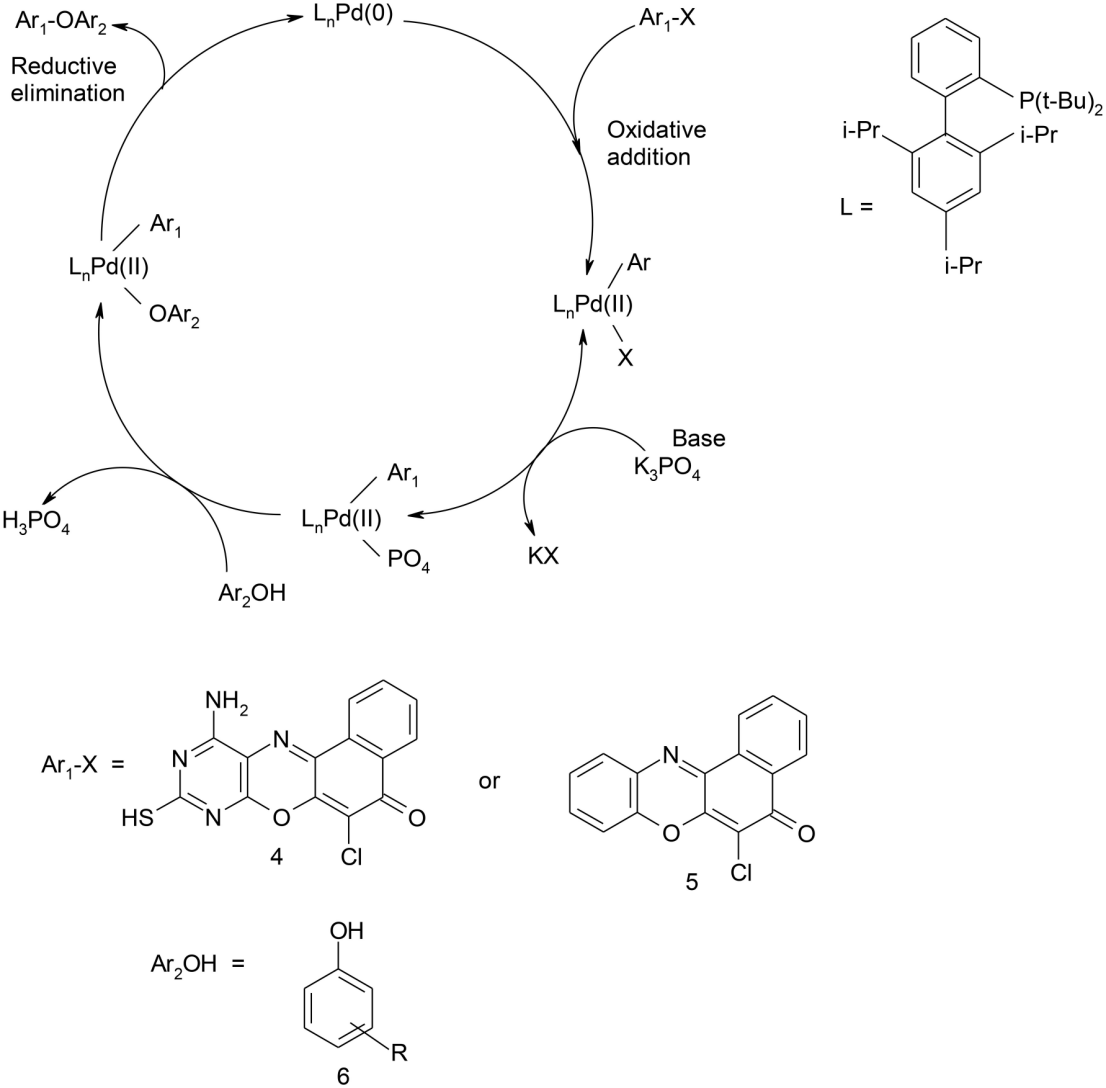
Catalytic cycle for C-O coupling of 11-amino-9-mercapto-8,10-diazabenzo[a]phenoxazin-5-one**/**6-chlorobenzo[a]phenoxazin-5-one and phenols.

Structural modifications of the phenoxazine ring systems using palladium based electron rich bulky aryldialkylphosphine ligand (t-BuXPhos) have given rise to 12 ether derivatives with significant synthetic yields (46–96%) as shown in Table 2. The two key intermediates in the syntheses were 11-amino-9-mercapto-6-chloro-8,10-diaza-5H-benzo[a]phenoxazin-5-one **4** and 6-chloro-5H-benzo[a]phenoxazin-5-one **5**, which were prepared by condensation of 4,5-diamino-6-hydroxyl-2-mercaptopyrimidine and 2-aminophenol with 2,3-dichloro-1,4-naphthoquinone respectively in a basic medium (Okafor, 1986). Reaction of compound **4** with various phenols in the presence of a catalyst system consisting of palladium (II) acetate and t–BuXPhos, afforded the ether derivatives **7a–f** in good yields (Table 2, entries 1–6). The reaction of compound **5**, under similar reaction conditions, gave compounds **8a–f** also in good yields (Table 2, entries 7–12). The success of these reactions is attributed to use of the electron-rich, sterically bulky aryldialkylphosphine, t-BuXPhos, as ligand. The results show that using the t-BuXPhos, an electron-rich phosphine ligand, can achieve oxidation of diazabenzo[a]phenoxazine **4** and benzo[a]phenoxazine **5** nuclei to palladium centers. There is a shift in the rate-determining step in the catalytic cycle of the reaction process from the oxidative addition of the diazabenzo[a]phenoxazine **4** and benzo[a]phenoxazine analog **5** to the reductive elimination, which subsequently led to the formation of compounds **7a–f** and **8a–f**, respectively (Scheme 3). Structure-activity relationship study shows that reaction of compounds **4** and **5** with electronically neutral phenol (C_6_H_5_OH) gave high yields (96 and 95%, entries 2 and 8) with compound **5** showing a lower yield. In addition, phenols bearing electron donating groups in para-position gave excellent yields (entries 1, 5, 7, and 11). The fact that these electron rich phenols are particularly good reaction partners showed that the presence of compounds **4** and **5** allows the delocalization of the electrons, which may have been generated in the transition state of the reductive elimination of the phenoxazine ether (Mann and Hartwig, 1997; Hartwig, 1998). Furthermore, the reaction of compounds **4** and **5**, with electron deficient phenol bearing chlorine at the para-position, gave a better yield with compound **4** (74%, entry 3) than with compound **5** (46%, entry 8). This is in agreement with the observation earlier made by other authors (Mann and Hartwig, 1997; Hartwig, 1998). When an electron rich compound is coupled with electron deficient phenolic compound, it gives higher yield than when lesser electron rich compound is coupled with electron deficient phenol compound. 11-Amino-9-mercapto-8,10-diazabenzo[a]phenoxazin-5-one **4** is more electron rich compound than 6-chlorobenzo[a]phenoxazin-5-one **5**, hence the difference in yields in this case. The highest yield was obtained when 1-naphthol was coupled with compound **4** (99%, entry 6). This may be attributed to the presence of the additional benzene ring in naphthol compound, which constituted increase in conjugation resulting in increased electron delocalization. Reaction of compound **5** with 1-naphthol, gave a lower yield (68%, entry 12). This may be because of difference in electronegativity of compounds **4** and **5**.

**Table 2:** Palladium-catalyzed phenoxazine ether derivatives formation from phenols^(a)^.

**Entry**	**Phenoxazine compounds**	**Phenols**	**Products**	**Reaction time (h)**	**% yields (%)**
**1**	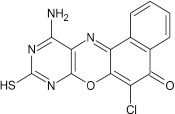	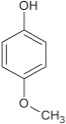	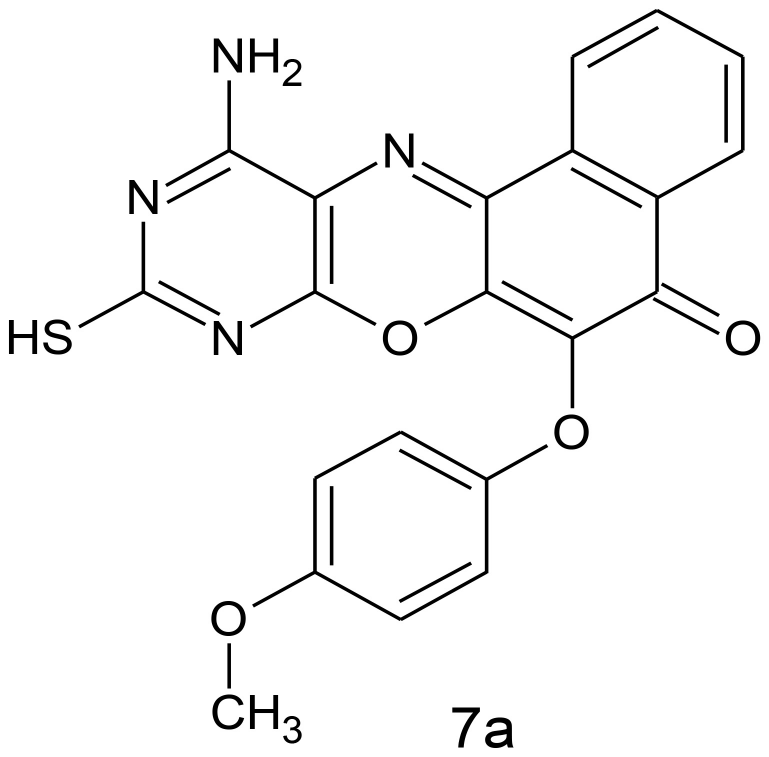	8	96
**2**	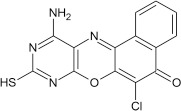	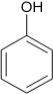	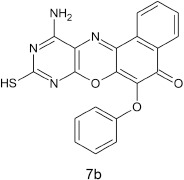	10	96
**3**	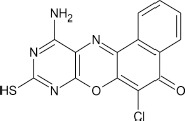	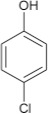	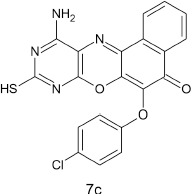	8	74
**4**	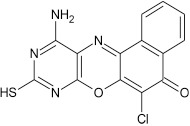	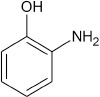	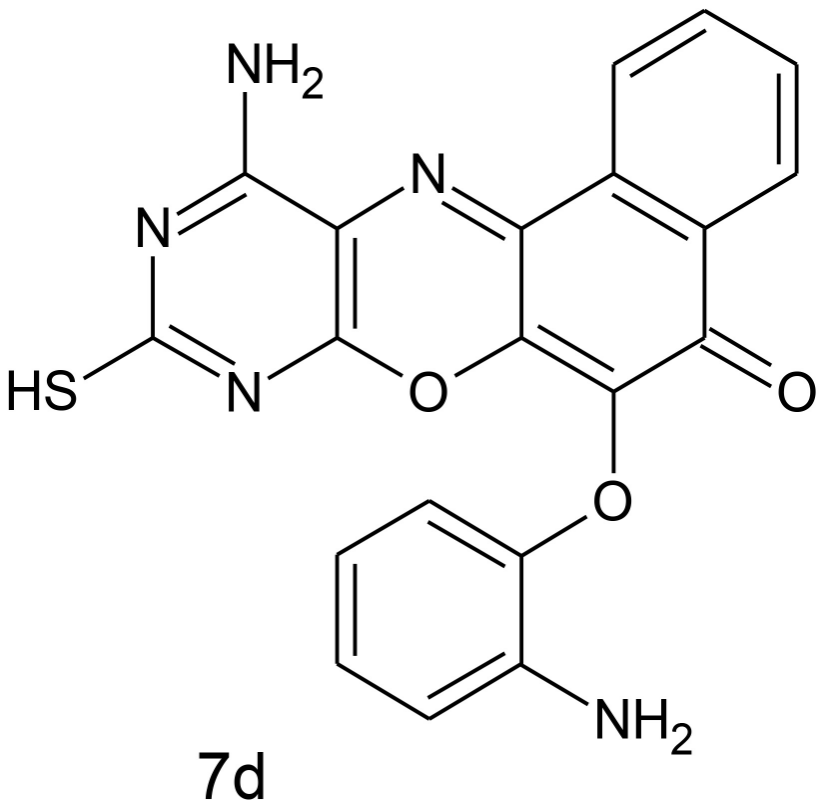	10	91
**5**	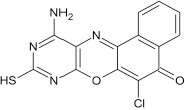	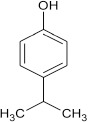	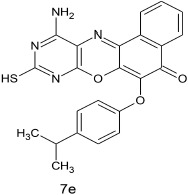	7	96
**6**	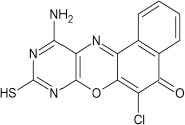	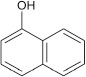	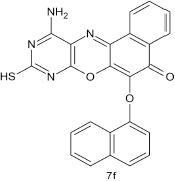	10	99
**7**	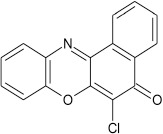	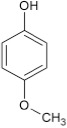	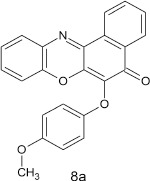	8	97
**8**	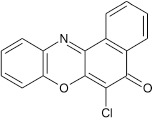	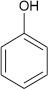	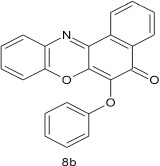	10	95
**9**	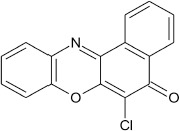	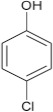	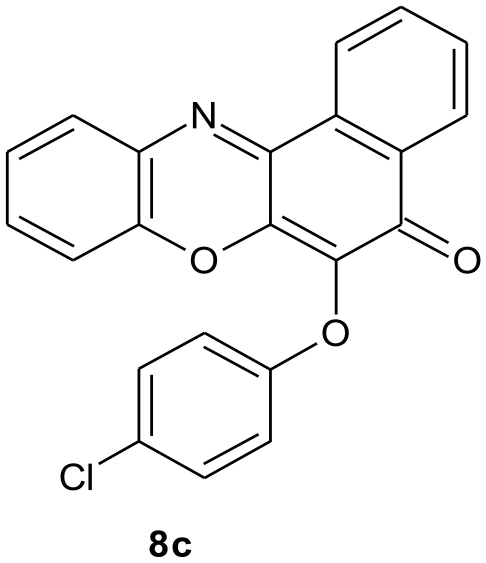	8	46
**10**	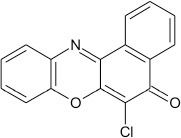	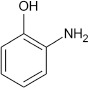	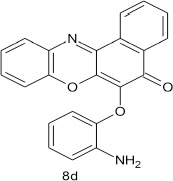	10	94
**11**	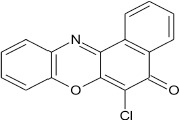	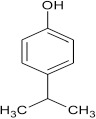	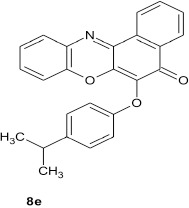	7	98
**12**	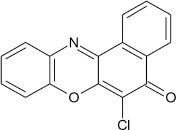	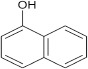	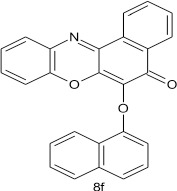	10	68

*(a) Reaction conditions: angular phenoxazine (1.0 equiv), phenol (1.2 equiv), K_3_PO_4_ (2.0 equiv), ligand (3.0 mol%), toluene (0.3mL), Reflux at 100–110°C, 6–10 h, palladium acetate (4.5 mg); Ligand used: 2-(di-ter-butylphosphino)biphenyl (t-BuXphos)*.

### Spectral characterization

**Scheme 4:**
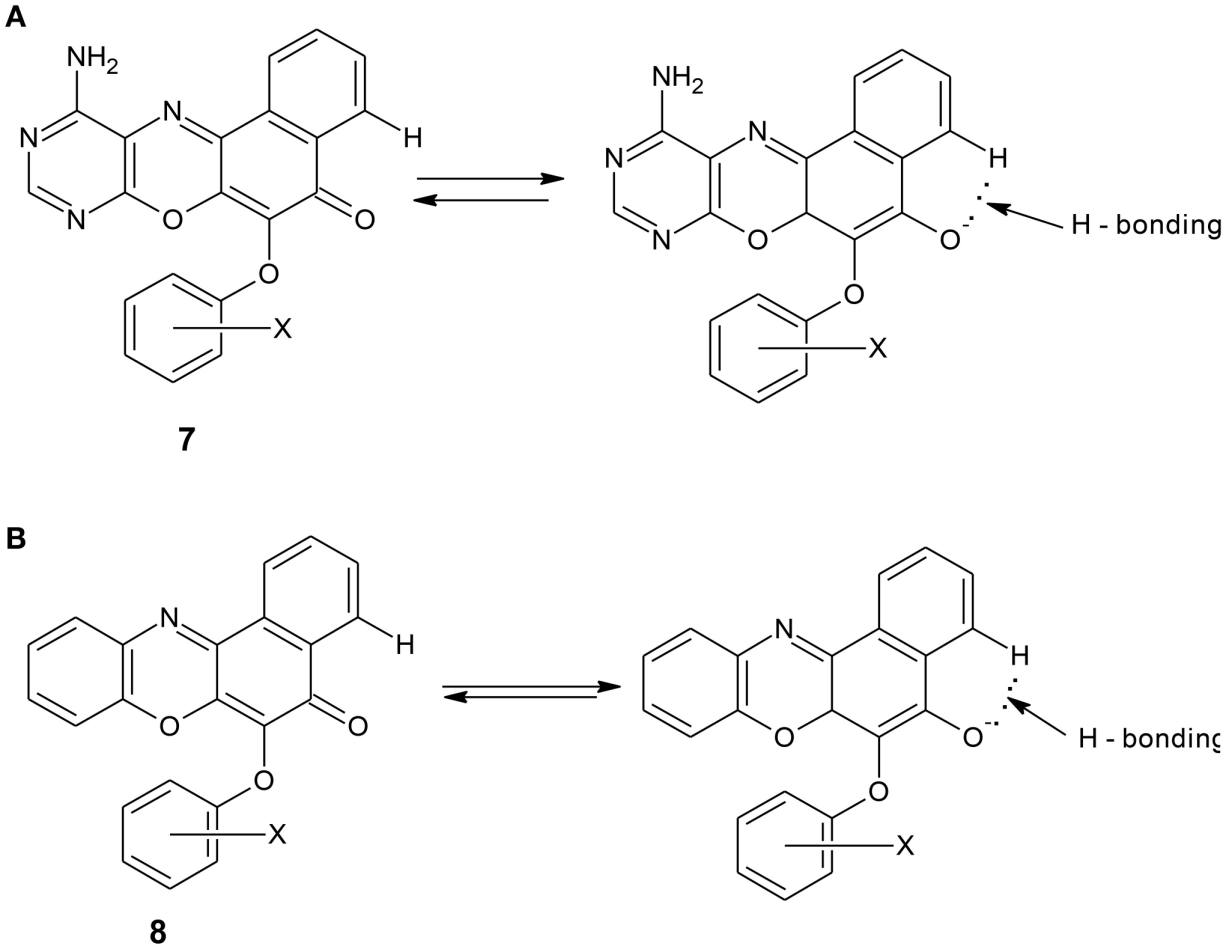
**(A)**: Intramolecular hydrogen bonding in diazaphenoxazine ether derivatives 7a. **(B)**: Intramolecular hydrogen bonding in benzophenoxazine ether derivatives 8a.

The spectral data are as shown in the experimental section. The electronic spectrum of some of the compounds showed peaks at 260–270 nm which is due to n–π^*^ transitions. The peaks at 251–258 nm were assigned to n–σ^*^ transitions. There were also peaks at 217–234 nm which are due to π-π^*^ transitions. The peaks at the longer wavelengths 451–490 nm are due to extended conjugation in the compounds, which resulted in increase in electron delocalization. Furthermore, all the compounds showed the characteristic absorption of phenoxazine ring systems at 385–390 nm. The compounds were further characterized using infrared data. The asymmetric and symmetric C–O–C stretching of ether compounds in IR spectra were observed at 1,208–1,269, and 1,058–1,065 cm^−1^, respectively, for all the ether phenoxazine derivatives. The peaks 1,626–1,641 cm^−1^ observed in all the spectra show the absorption of C = O which forms O-hydroxyl aryl intramolecular hydrogen bonding (Schemes 4a,b). This may account for the deliquescent nature of the synthesized compounds. The intramolecular hydrogen bonding gave peak observed at 3,163–3,197 for compounds **8a–f**. This peak was shown for compounds **7a–f** at 3,183–3,205 cm^−1^. The peak 3,183–3,205 cm^−1^ was also attributed to N-H stretch of primary aromatic amine in compounds **7a–f**. Other peaks are in agreement with the assigned structure (Experimental). The NMR spectra further supported the assigned structures. For example, the ^1^H NMR spectra of the diazaphenoxazine ether derivatives **7a–f** showed singlet peak at δ 2.53–3.56 ppm assigned to SH proton. This peak was not seen in the spectra of the benzo analog **8a–f**. Other peaks in the NMR spectra as shown in the experimental section are in agreement with the assigned structures. For the ^1^H NMR spectra of compounds **7a–f** and **8a–f**, see the Supplementary Materials.

### *In-silico* physicochemical evaluation

The physicochemical properties such as molecular weight (MW), Partition coefficient value (Log P), which indicates the Lipophilicity of the ligand; number of hydrogen bond donor (HBD) and number of hydrogen bond acceptor (HBA), total polar surface area (TPSA), number of rotatable bonds (NoRB), and volume for the ligands were obtained as shown in Table 3. For a drug molecule to be orally bioavailable in the systemic circulation, Lipinski's rule of five should apply: “the drug must have molecular weight value of ≤500, hydrogen bond donor ≤5, hydrogen bond acceptor ≤10, and partition coefficient (Log P) value ≤5.” From the results in Table 3, the synthesized compounds are in agreement with the Lipinski's rule of five since there was no violation of more than one parameter studied. In addition, the Polar Surface Area (PSA), which reflects the ligand hydrophilicity, is very vital in protein-ligand interaction. Veber et al. (2002) showed that 10 or less rotatable bonds and polar surface area, PSA ≤140 Å^2^ would have a high probability of good oral bioavailability in rats. Compounds **8a–f** were found to have PSA ≤90 Å^2^. According to van de Waterbeemd et al. (1998), they can cross the BBB and penetrate the CNS, and hence, they are potential candidates for the treatment of cerebral malaria.

**Table 3:** *In-silico* physicochemical properties.

**Comp**.	**MW**	**Log P**	**HBD**	**HBA**	**nViolation**	**TPSA**	**NoRB**
**7a**	418.43	4.20	2	8	0	113.38	3
**7b**	388.41	4.15	2	7	0	104.14	2
**7c**	422.85	4.82	2	7	0	104.14	2
**7d**	403.42	3.58	4	8	0	130.17	2
**7e**	430.49	5.66	2	7	1	104.14	3
**7f**	438.47	5.30	2	7	1	104.14	2
**8a**	369.38	5.49	0	5	1	61.57	3
**8b**	339.35	5.43	0	4	1	52.34	2
**8c**	373.80	6.11	0	4	1	52.34	2
**8d**	354.37	4.87	2	5	0	78.36	2
**8e**	381.43	6.95	0	4	1	52.34	3
**8f**	389.41	6.59	0	4	1	52.34	2
**4**	330.76	3.07	2	6	0	94.91	0
**5**	281.70	4.36	0	3	0	43.10	0
Ciprofloxacin	331.35	−0.70	2	6	0	74.57	3

*The Red colored values imply above normal range (log P value ≤ 5), recommended by Lipinski's rule*.

### Docking studies

**Figure 1:**
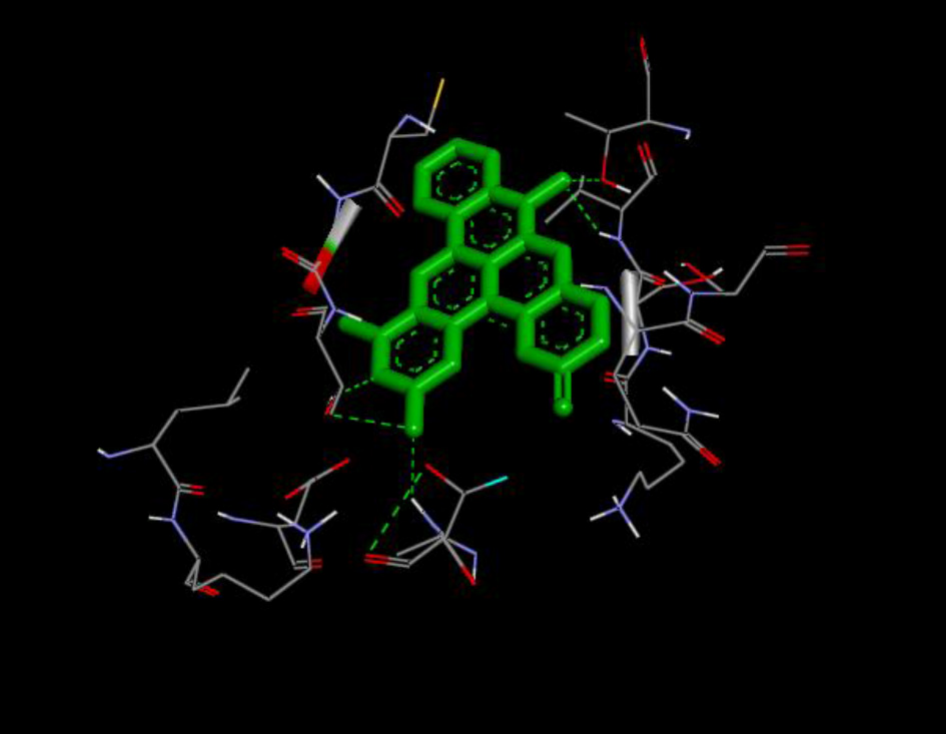
Binding mode of compound 7c with 2VF5.

**Figure 2:**
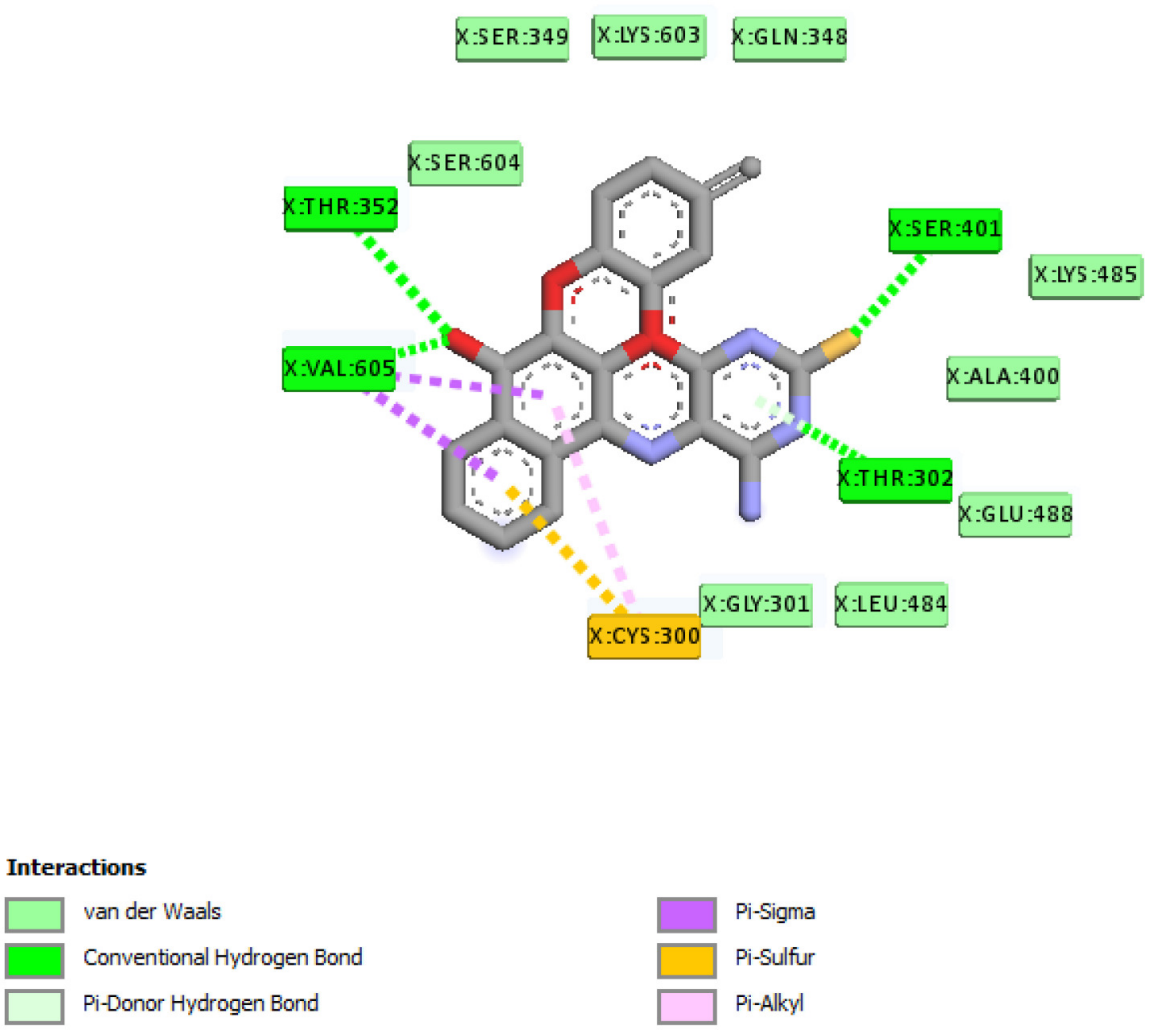
Different chemical interactions of compound 7c with the amino acid residues of 2VF5.

**Figure 3:**
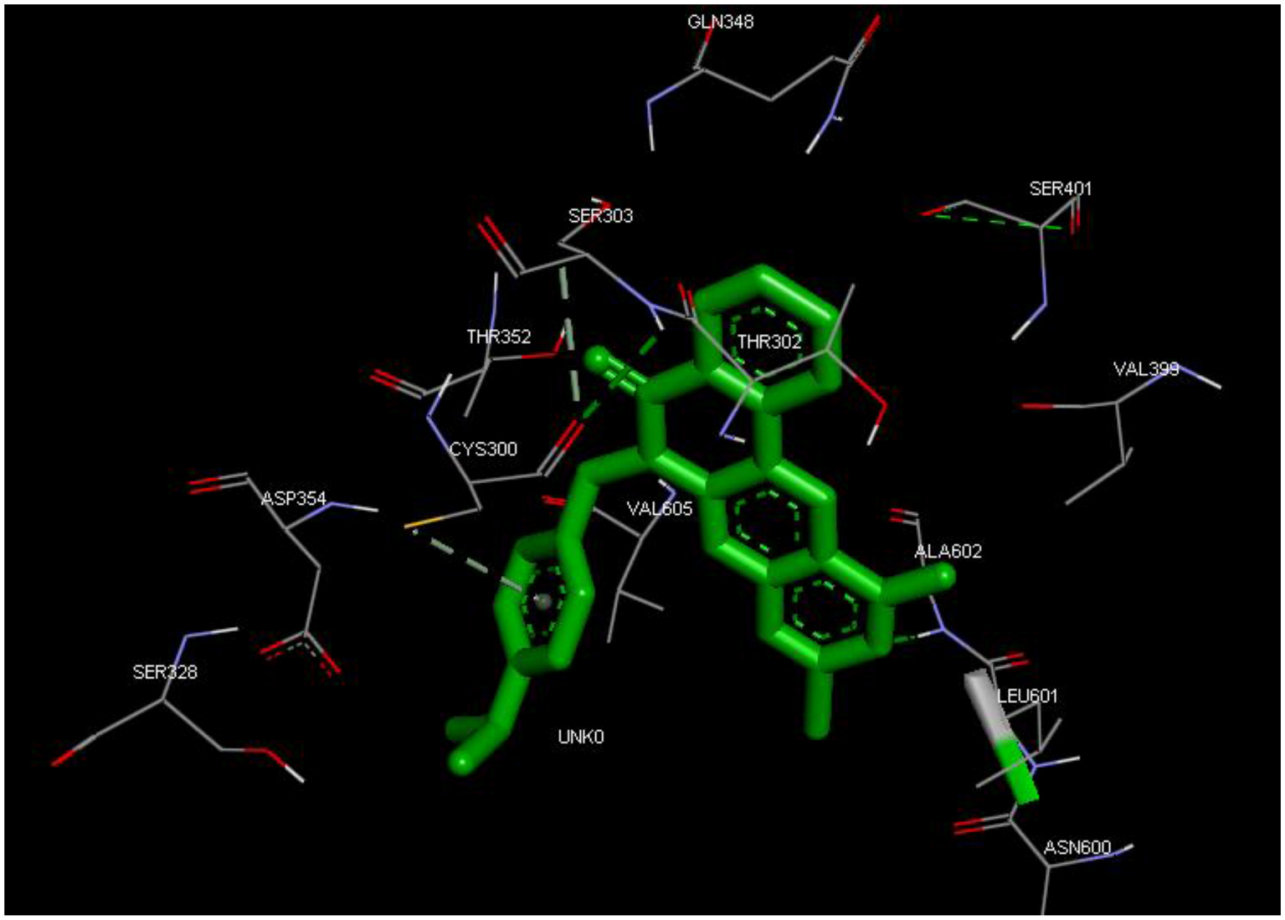
Binding mode of compound 7e with 2VF5, showing the amino acid residues.

**Figure 4:**
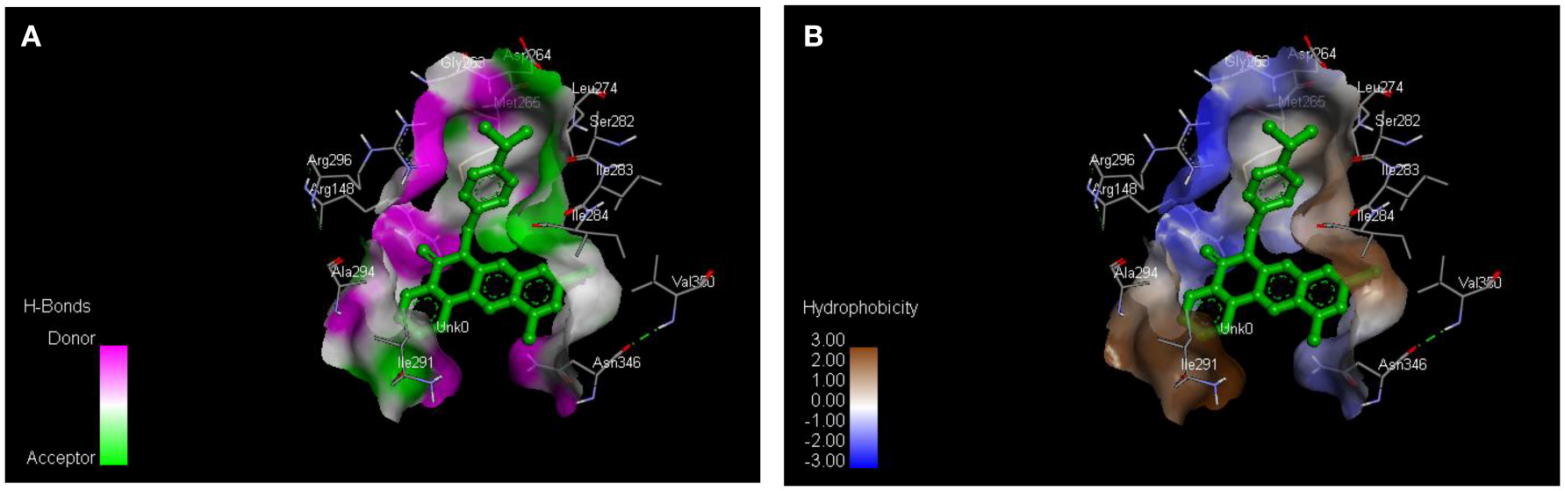
Compound 7e in the binding cavity of 2VF5, showing **(A)** H-bonds and **(B)** hydrophobicity regions of the binding site.

From the molecular docking results, modifications of compounds **4** and **5** gave rise to compounds (**7a–e**) and (**8a–e**), respectively, which showed better binding affinities with the targets. However, there was no significant difference in the binding affinities of the modified compounds (**7a, b, d, f**, and **8a–d, f**). In addition, there were no significant binding affinities with the receptors between the diazabenzo[a]phenoxazin-5-one and benzo[a]phenoxazin-5-one derivatives. It was also observed that compounds **7c, 7e**, and **8e** had best interactions with the three target proteins as seen in their appreciable increase in binding affinities (Table 4). This underscores the usefulness of these compounds as potential antimicrobial agents since they can bind to multiple targets in the organism, which will result in the inhibition of several biochemical pathways essential for the survival of the organism and their consequent death. Although all the three target proteins showed good interactions with the compounds, the protein receptor, **2VF5** gave best interaction. Closer docking studies of compounds **7c** and **7e** with the receptor, **2VF5** have been demonstrated (Figures 1–4) in order to gain further insight. Figure 1 shows the binding mode of **7c** with 2VF5; Figure 2 demonstrates its interactions and the nature of interaction with the amino acid residues of the receptor. Figure 3 demonstrates the binding mode of compound **7e** with **2VF5**, and the types of amino acid residues involved in the interactions, whereas Figure 4 showing compound **7e** in the binding cavity of **2VF5**, illustrates H-bonds (A) and hydrophobicity (B) regions of the binding site. Compound **7c** interacted with the following active amino acid residues of **2VF5**: THR:352, THR:302, VAL:605 AND SER:401 forming H-bonds; VAL:605 forming pi-sigma bonds and CYS:300 forming pi-sulfur and pi-alkyl bonds (Figure 2).

**Table 4:** Binding affinity of different compounds with selected bacteria target proteins.

**Compound**	**Binding energy**, Δ**G (kcal/mol)**		
	**2VF5**	**1KE4**	**3JUV**
**4**	−7.8	−8.0	−8.2
**5**	−7.4	−7.9	−8.5
**7a**	−8.3	−8.5	−7.6
**7b**	−8.3	−8.7	−9.0
**7c**	−9.2	−9.1	−9.1
**7d**	−8.1	−9.1	−9.2
**7e**	−9.5	−9.3	−9.3
**7f**	−8.0	−8.3	−8.5
**8a**	−8.7	−8.2	−8.6
**8b**	−8.7	−8.0	−8.5
**8c**	−9.0	−8.5	−8.3
**8d**	−8.6	−8.7	−9.0
**8e**	−8.7	−8.6	8.3
**8f**	−9.5	−9.3	−9.3
Ciprofloxacin	−7.8	−7.5	−7.1
Ketoconazole	−8.2	−8.7	−8.6

### QSAR analysis results

Predicted MIC (μg/ml) = 5.68704 + 1.05391 × AM1_dipole + 5.91138 × a_acc + 10.48717 × a_don −1.98295 × b_rotN −5.02794 × logP(o/w)

−1.36589 × rings − 0.45882 × TPSA + 0.25853 × Weight − 2.69962 × b_ar

Regression coefficient (*N* = 50, *R*^2^ = 0.7400, *R*^2^__(predicted)_ = 0.5475).

The above-mentioned equation clearly showed that the molecular descriptors, number of rotatable bonds, logP (o/w), number of rings, total polar surface area, and number of aromatic bond showed negative correlation with respect to the biological activity. On the other hand, dipole moment, number of H-donors, number of H-acceptors, number of aromatic bond, and molecular weight showed positive correlation with the biological activity.

**Figure 5:**
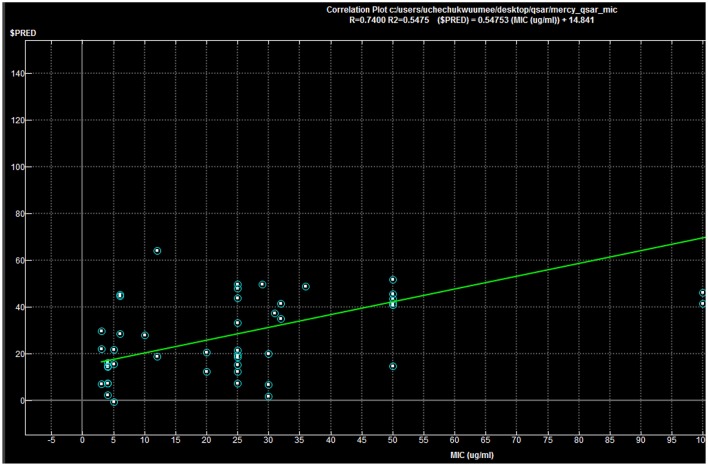
Correlation plot: *R* = 0.7400, *R*^2^ = 0.5475; ($PRED) = 0.54753(MIC) + 14.841.

**Figure 6:**
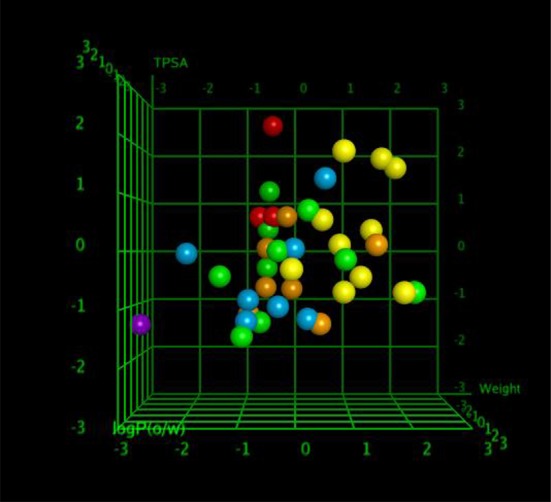
The 3D scatter plot of three major descriptors.

QSAR model analysis includes the calculation of cross-validated squared correlation coefficient (*R*^2^) for internal validation and the predictive squared correlation coefficient (*R*^2^__predicted_) for external validation. Here, in this case, the *R*^2^ was 0.7400, and R^2^__predicted_ was 0.5475, which undoubtedly showed the true predictability of the model and that it was not by mere chance. Graphical plot between experimental and predicted activities MIC (μg/ml) of the training and test set compounds are represented in Figures 5, 6 also showed the 3D scatter plot of the three major descriptors (molecular weight, logP (o/w) and TPSA) used.

Each point in the MOE Window corresponds to a molecule, and is colored according to the molecules MIC-value.

## Conclusion

Structural modifications of phenoxazine nucleus gave rise to new chemical moieties of high yields. These base mediated coupling reactions proceed excellently in toluene in excellent yields and require short reaction time. The physicochemical evaluations showed that the compounds exhibit strong drug-likeness and could be bioavailable in the systemic circulation when orally administered. The molecular docking studies performed have confirmed that the compounds fit into the binding pockets of the bacterial target proteins and demonstrated an excellent binding affinity. The hydrogen bonding and other hydrophobic interactions between the synthesized compounds and the receptors were very important for the successful protein target inhibitions observed. These compounds could be used to treat multi-drug resistance infections caused by bacteria and fungi.

## Author contributions

ME and UO designed the synthetic work. ME and OO carried out the synthesis and characterization of all the compounds. ME, OO, and SO were all involved in the *in vitro* biological assays. SO carried out the computational experiments and performed the acquisition, analysis, and interpretation of data. SO, ME, and OO wrote the manuscript and UO, EG-N, and FI evaluated the integrity of every section of the manuscript. All the authors read the manuscript and made scientific contributions in revising it.

### Conflict of interest statement

The authors declare that the research was conducted in the absence of any commercial or financial relationships that could be construed as a potential conflict of interest.
