# Supplementary material for: Angular Phenozaxine Ethers as Potent Multi-microbial Targets Inhibitors: Design, Synthesis, and Molecular Docking Studies
Source: Front Chem. 2017 Nov 28;5:107. doi: 10.3389/fchem.2017.00107 (PMC5712349; doi:10.3389/fchem.2017.00107)
Supplement: Supplementary file 1 [file DataSheet1.DOC]

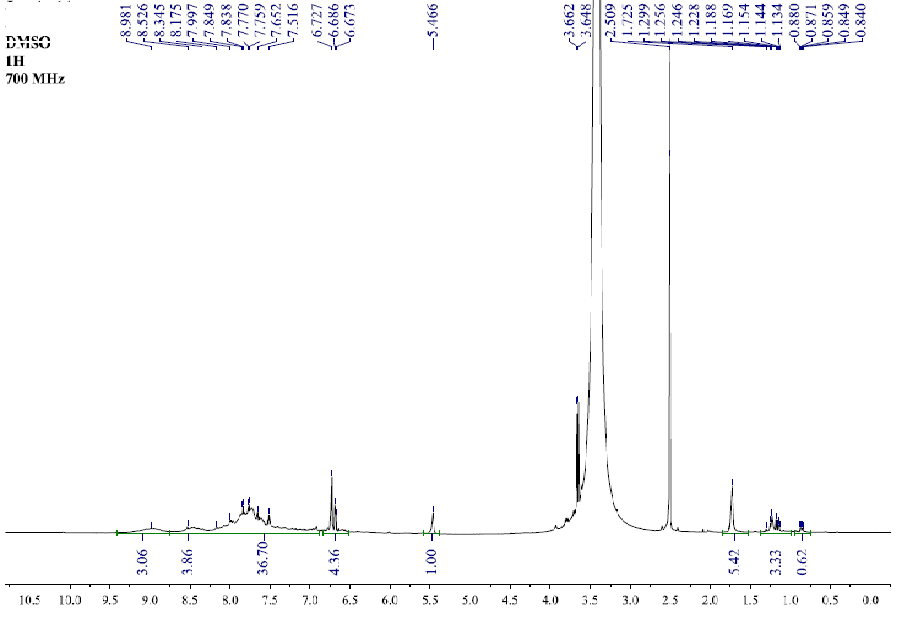


Figure 1: ^1^H NMR of compound 7a


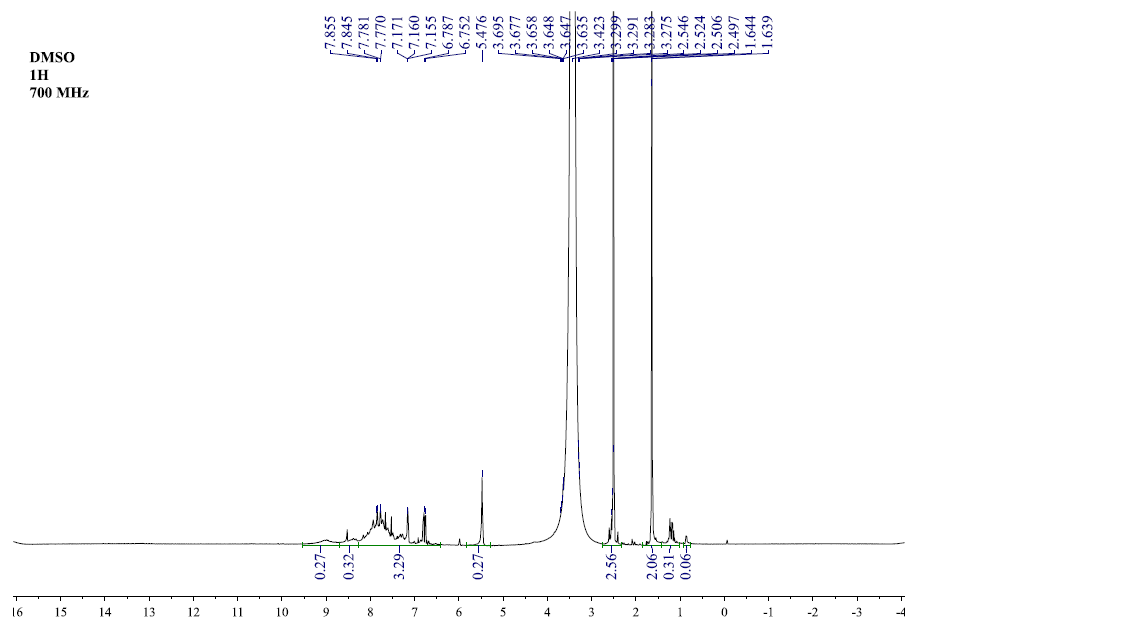


Figure 2: ^1^H NMR of compound 7b


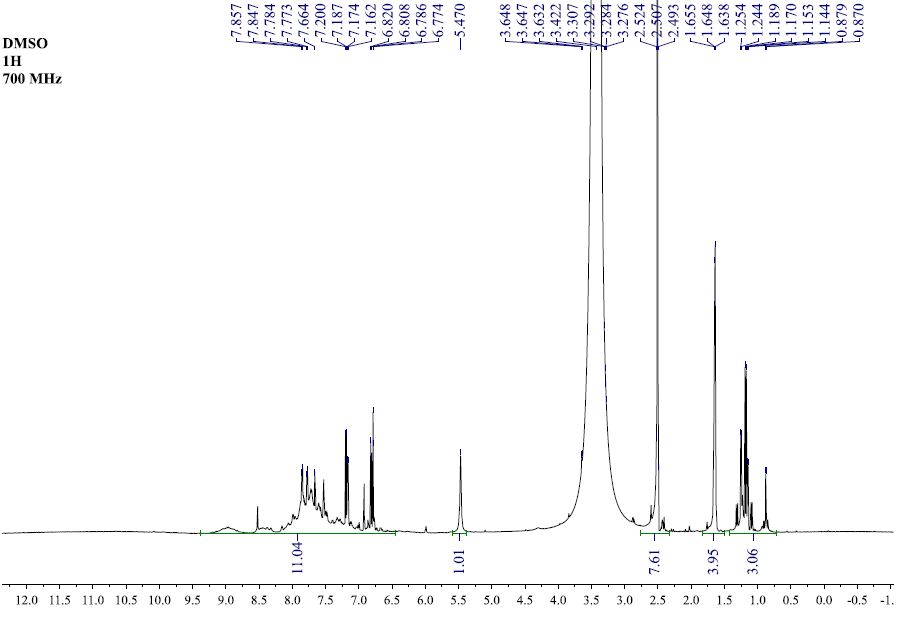


Figure 3: ^1^H NMR of compound 7c


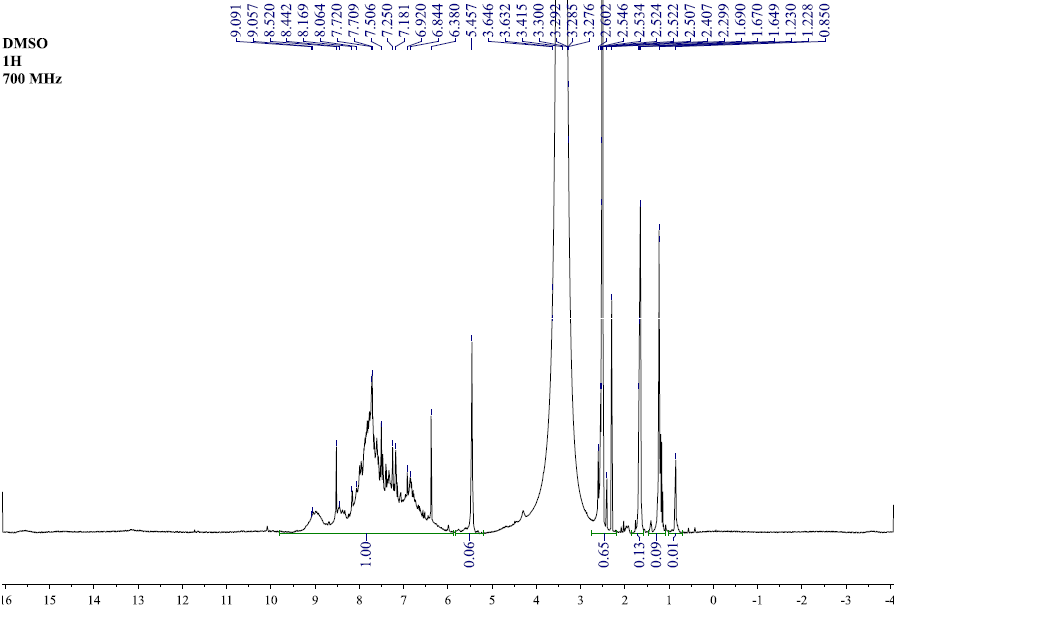


Figure 4: ^1^H NMR of compound 7d


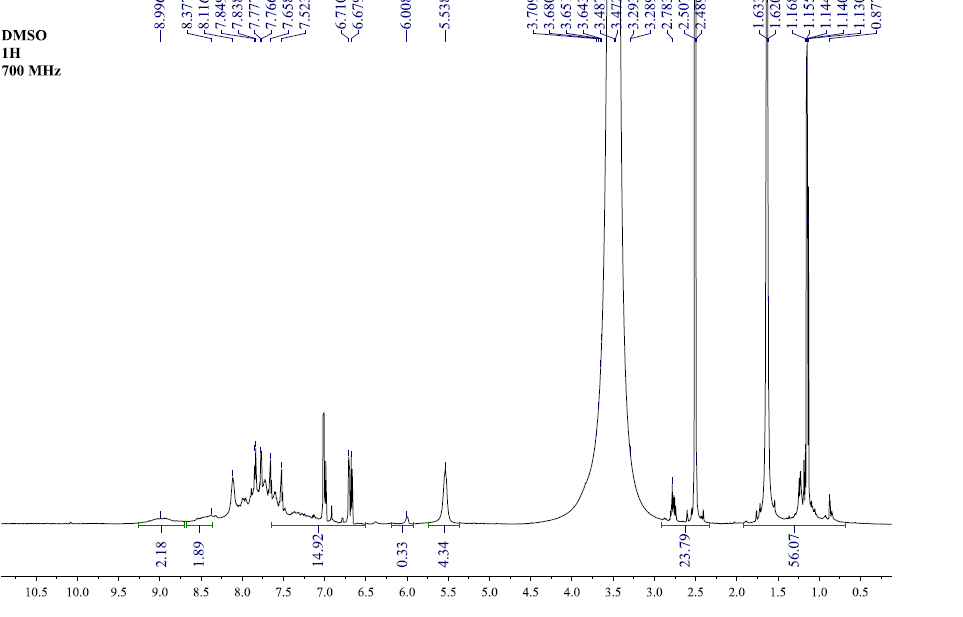


Figure 5: ^1^H NMR of compound 7e


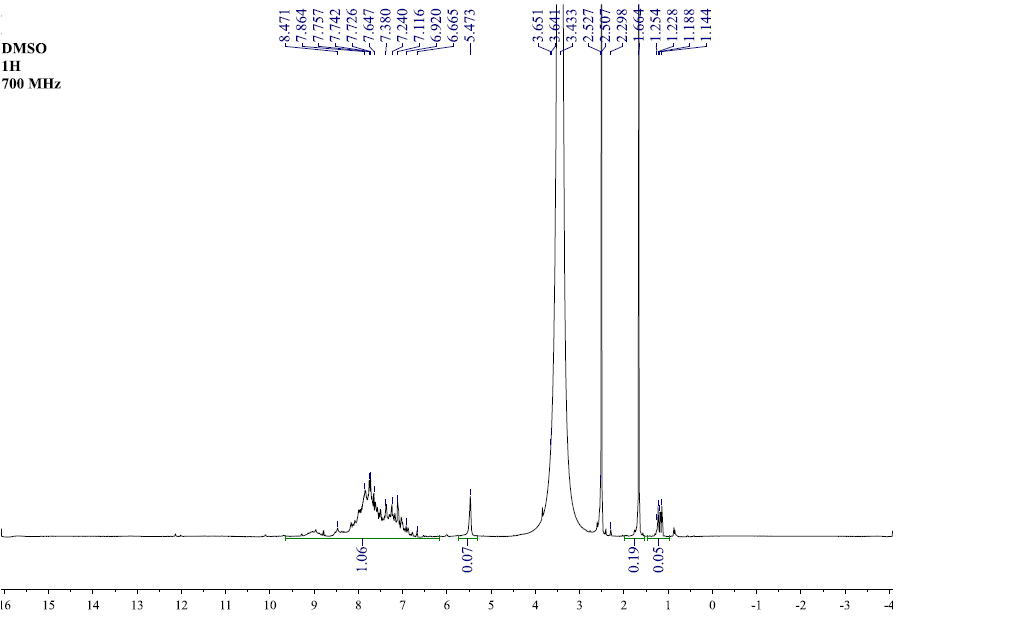


Figure 6: ^1^H NMR of compound 7f


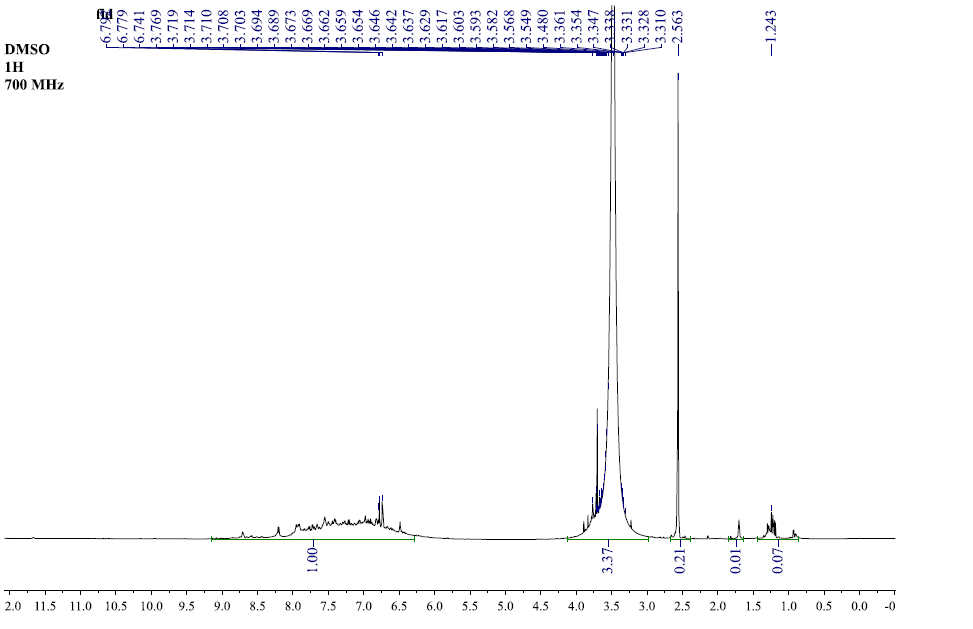


Figure 7: ^1^H NMR of compound 8a


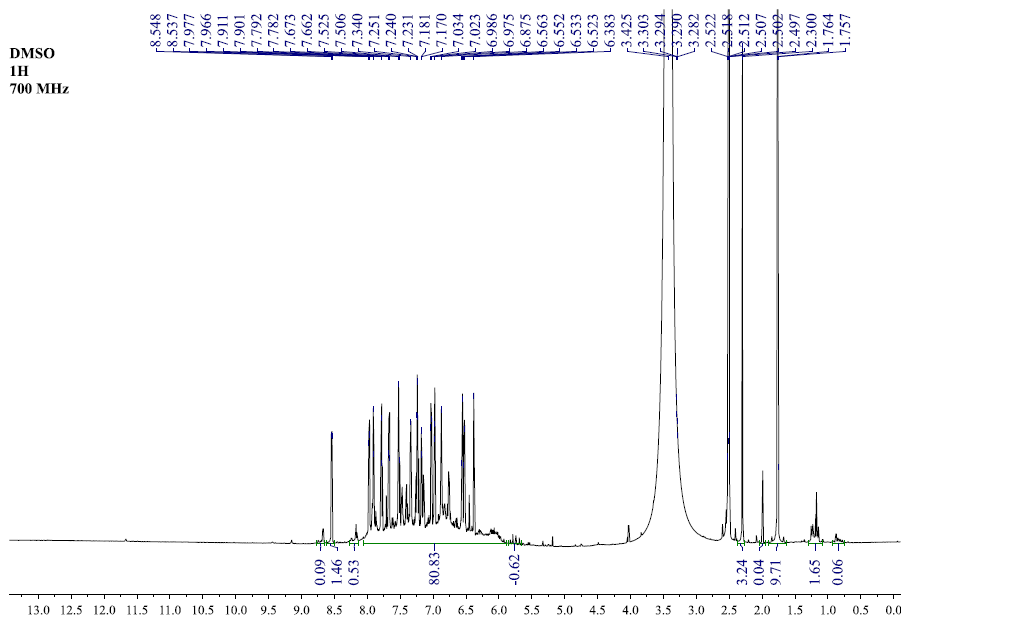


Figure 8: ^1^H NMR of compound 8b


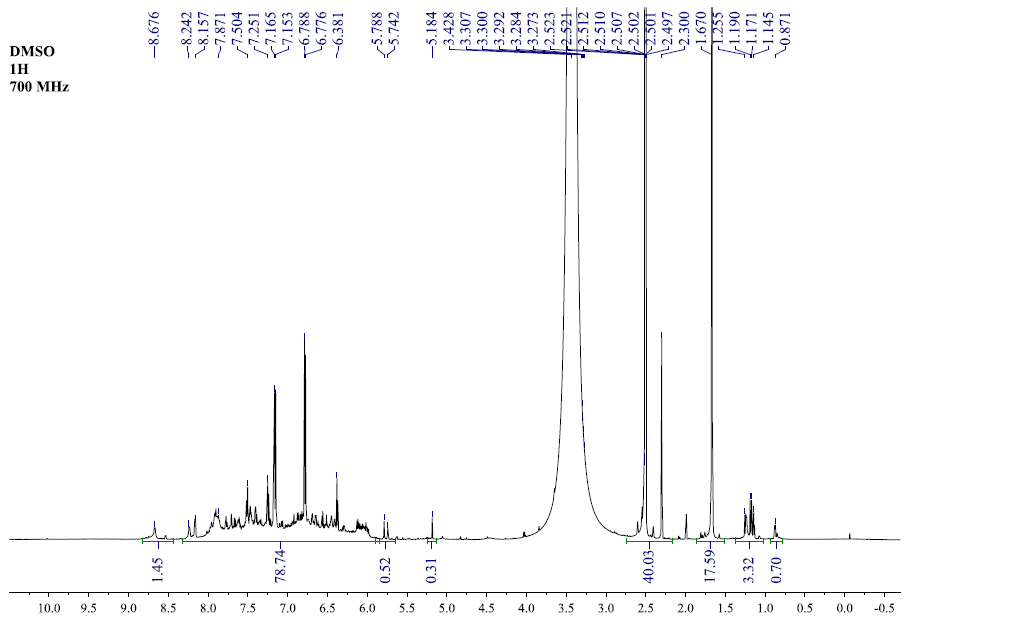


Figure 9: ^1^H NMR of compound 8c


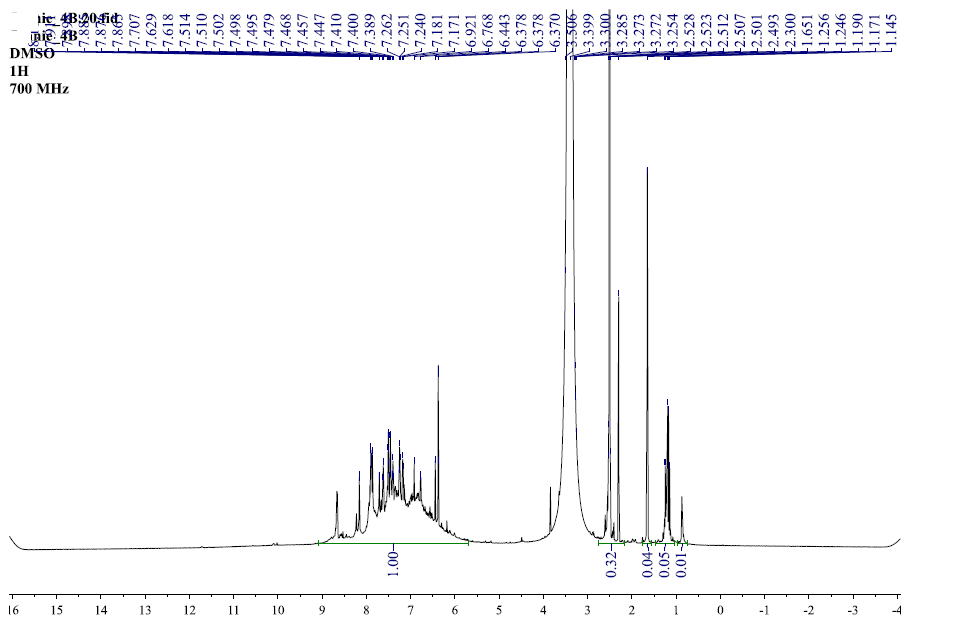


Figure 10: ^1^H NMR of compound 8d


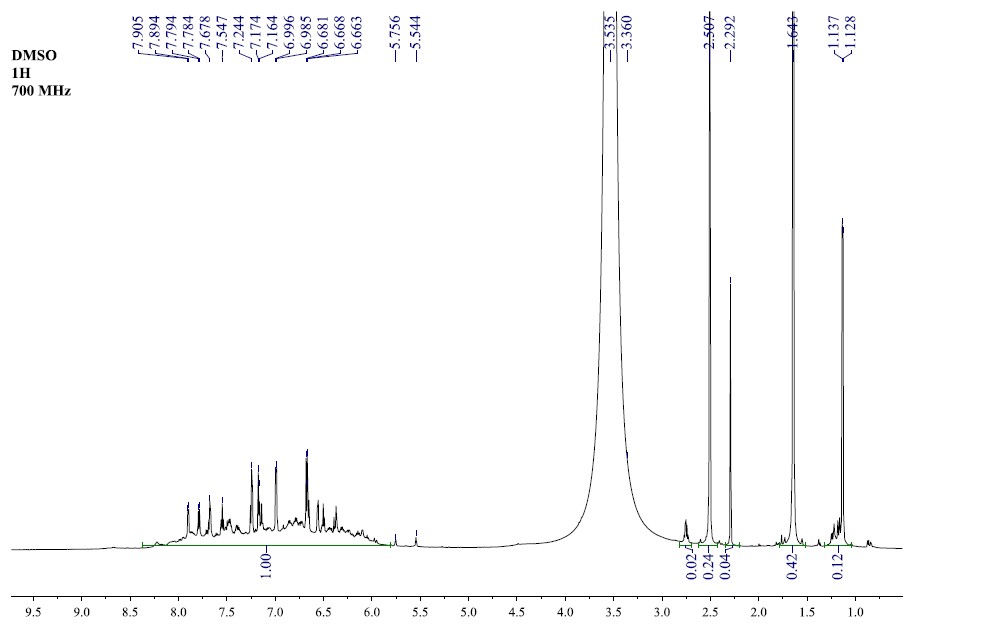


Figure 11: 1H NMR of compound 8e


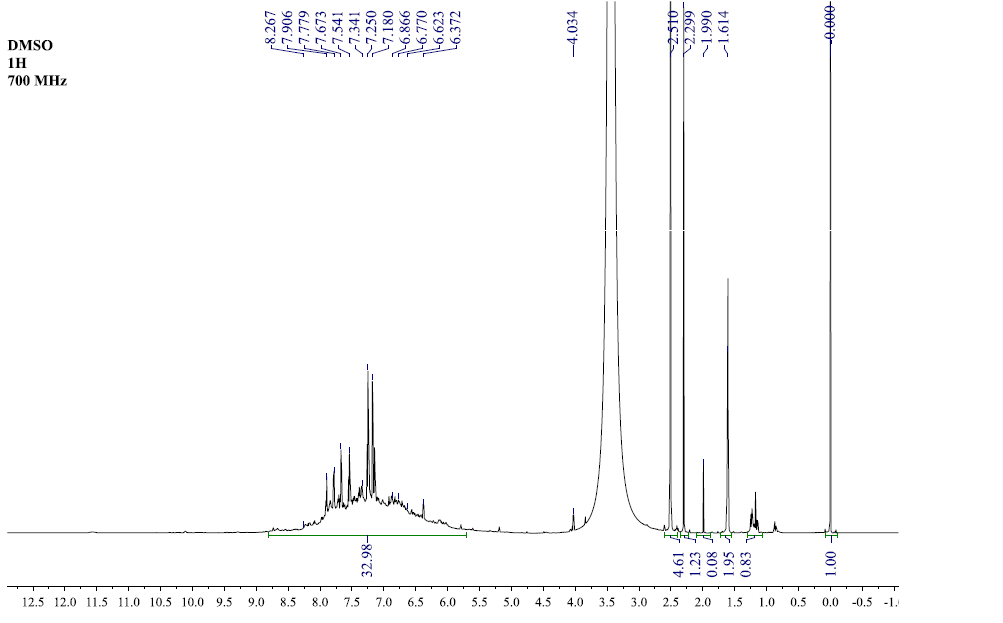


Figure 12: 1H NMR of compound 8f
